# Supplementary material for: Complex‐centric proteome profiling by SEC‐SWATH‐MS
Source: Mol Syst Biol. 2019 Jan 14;15(1):e8438. doi: 10.15252/msb.20188438 (PMC6346213; doi:10.15252/msb.20188438)
Supplement: Supplementary file 8 — Dataset EV7 [file MSB-15-e8438-s008.zip › feature_plots_string/P00156.pdf]

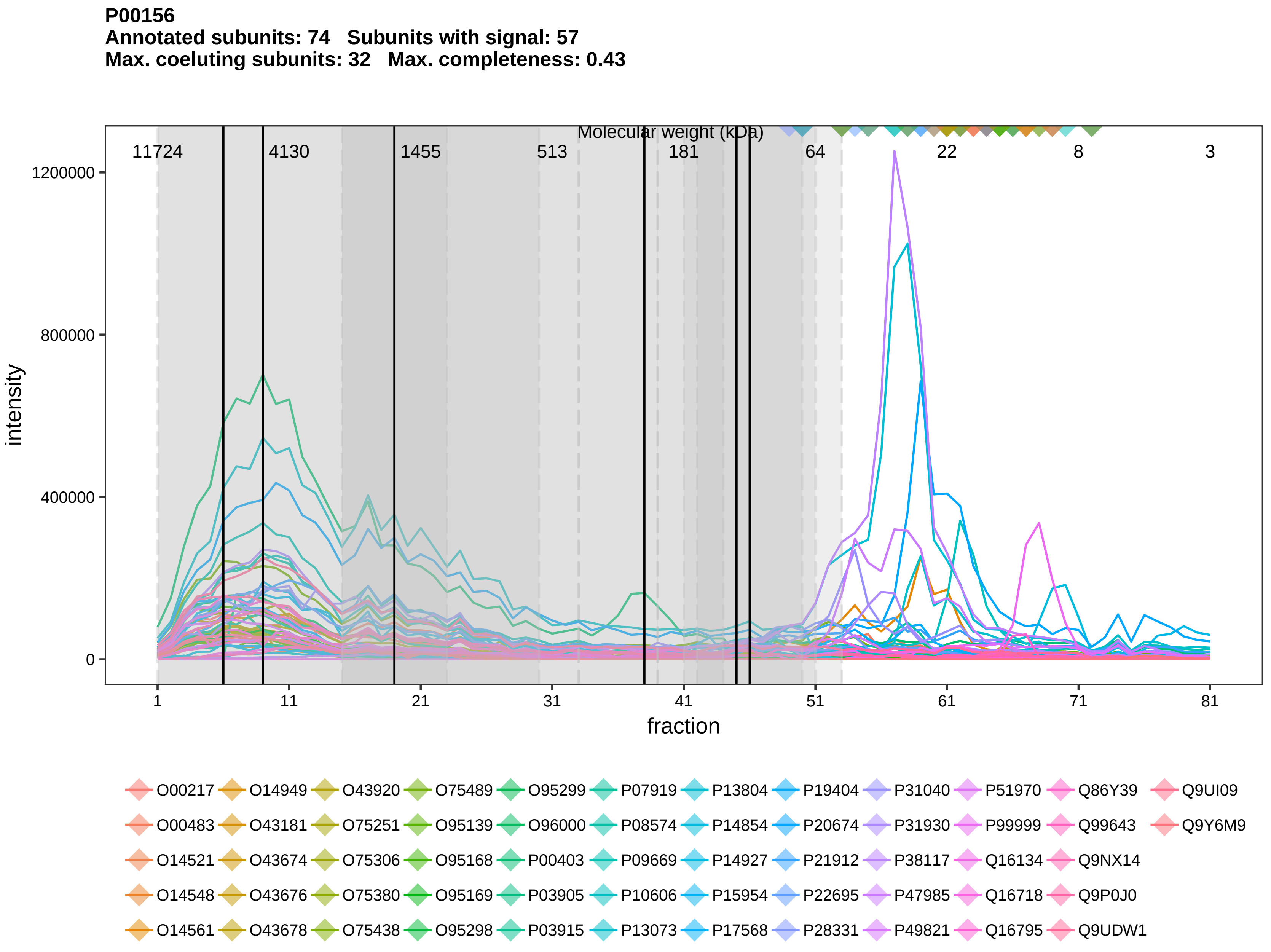

P00156
Annotated subunits: 74 Subunits with signal: 57
Max. coeluting subunits: 32 Max. completeness: 0.43
Molecular weight (kDa)
11724
4130
1455
513
181
64
22
8
3
intensity
1200000
800000
400000
0
fraction
1
11
21
31
41
51
61
71
81
O00217
O00483
O14521
O14548
O14561
O14949
O43181
O43674
O43676
O43678
O43920
O75251
O75306
O75380
O75438
O75489
O95139
O95168
O95169
O95298
O95299
O96000
P00403
P03905
P03915
P07919
P08574
P09669
P10606
P13073
P13804
P14854
P14927
P15954
P17568
P19404
P20674
P21912
P22695
P28331
P31040
P31930
P38117
P47985
P49821
P51970
P99999
Q16134
Q16718
Q16795
Q86Y39
Q99643
Q9NX14
Q9P0J0
Q9UDW1
Q9UI09
Q9Y6M9
